# Dr. C. G. Polk and the “Hospital Gazette”

**Published:** 1877-11-20

**Authors:** 


Dr. C. G. Polk and the *Hospital Gazette*.—It appears that Dr. Polk, of Philadelphia, is charged by the *Hospital Gazette* with being an "Irregular" and connected with a bogus Medical College in Philadelphia. He is also charged with contributing a paper on *Glycirite of Kephaline*, to a number of journals, having in view the advertising of the article for his own pecuniary benefit. We published an article from Dr. Polk in our June number, on the subject named, but had not noticed that the same article was elsewhere published. Subsequently we received an advertisement of the drug alluded to, for insertion in our journal, accompanied with the statement that the paper previously published having benefitted him, he felt it due to us that he furnish an advertisement of the remedy, which he did, and which may be seen in our advertising department. This does not seem to indicate a desire to secure the benefit of an advertisement without an equivalent.

He also writes a communication, denying in strong terms, the charge of being an "Irregular," or of being a Professor in a bogus college, and further vindicates himself against plagiarism, and the other charges preferred.

We, of course, cannot, at this writing, say to what extent, if any, the above charges may have foundation in truth. We hope that Dr. P. will be able fully to exculpate himself from them all.

